# Defect Inspection of Flip Chip Solder Bumps Using an Ultrasonic Transducer

**DOI:** 10.3390/s131216281

**Published:** 2013-11-27

**Authors:** Lei Su, Tielin Shi, Zhensong Xu, Xiangning Lu, Guanglan Liao

**Affiliations:** 1 State Key Laboratory of Digital Manufacturing Equipment and Technology, Huazhong University of Science and Technology, Wuhan 430074, China; E-Mails: lei_su2009@hust.edu.cn (L.S.); tlshi@hust.edu.cn (T.S.); zhensongxu@gmail.com (Z.X.); 2 Jiangsu Normal University, Xuzhou 221116, China; E-Mail: lxnam89@163.com

**Keywords:** flip chip, ultrasonic inspection, support vector machine, defect inspection

## Abstract

Surface mount technology has spurred a rapid decrease in the size of electronic packages, where solder bump inspection of surface mount packages is crucial in the electronics manufacturing industry. In this study we demonstrate the feasibility of using a 230 MHz ultrasonic transducer for nondestructive flip chip testing. The reflected time domain signal was captured when the transducer scanning the flip chip, and the image of the flip chip was generated by scanning acoustic microscopy. Normalized cross-correlation was used to locate the center of solder bumps for segmenting the flip chip image. Then five features were extracted from the signals and images. The support vector machine was adopted to process the five features for classification and recognition. The results show the feasibility of this approach with high recognition rate, proving that defect inspection of flip chip solder bumps using the ultrasonic transducer has high potential in microelectronics packaging.

## 1. Introduction

Flip chip technology combined with solder bumps interconnections is applied widely in electronic device manufacturing. With the tendency of flip chips toward ultra-fine pitch and high density together with the new requirements of packaging materials such as lead free and low-K, defects and failures happen more easily in flip chips [1–3], and the inspection becomes more critical and difficult. Traditional approaches for flip chip solder bump assessment include electrical testing, visual inspection, X-ray inspection, infrared thermography, and laser-ultrasound interferometer techniques. They are often insufficient due to their particular disadvantages. For instance, electrical testing inspects the solder bumps by measuring changes in electrical resistance and impedance [4]. Probes are contacted with the pre-designed test pads and a small electrical current passes through the chips to check each solder bump. This test is time consuming and expensive for complex boards, and any type of mechanical contact may make the defective bumps pass this testing [5]. Automated optical inspection (AOI) cannot detect solder bump defects hidden in the packaging directly, although it performs well in inspecting the solder bumps located on the edge of the die [6–8]. The laser ultrasound and interferometer techniques are developed to monitor the transient out-of-plane displacement response of the electronic package under pulsed laser excitation [9]. This is effective to detect solder bumps with large diameter and pitch. Infrared thermography is also used for solder bump inspection [10]. Chai *et al.* [11] utilized the hot spots in thermography to detect solder bumps when an electrical current passed through daisy chained chips. It is suitable for voids and partial cracks detection. X-ray radiography applies transmission of X-rays through the chips and substrates to perform defect inspection. The internal material has distinctly different X-ray absorbency [12], thus the variances in the shape and thickness of solder bumps can be revealed by X-ray images, and a fuzzy rule-based system was proposed to inspect the short circuits and defective solder bumps by use of the X-ray images [13]. However, the harmful radiation of X-ray equipment is unavoidable. Ultrasonic inspection is used extensively now [14], and scanning acoustic microscopy (SAM) has gained wide acceptance. It employs an ultrasonic source to scan across the sample surface, and uses the reflected waves to indicate the internal conditions of the components [15]. Semmens *et al.* utilized high frequency acoustic microscopy to analyze flip chip failures [16,17]. Zhang *et al.* [18,19] applied a sparse signal representation method to improve scanning acoustic microscopy and evaluate microelectronic packages. Normally the SAM results are dependent on the operators' experience, which makes it unreliable for inspecting flip chips with fine pitch and high density because of the inevitable visual fatigue.

Artificial neural network (ANN) is a system that consists of an interconnected group of artificial neurons that adaptively changes its structure through a training process [20,21]. It has predictive capability able to learn patterns from real data, together with some drawbacks such as slow convergence speed, poor stability and easily falling into local extrema. The support vector machine (SVM) learning method, which can overcome the problem of the local extremum existing in ANN and deal with small sample data with good generalization performance, has been promulgated as effective for pattern recognition [22]. Yun *et al.* [23] inspected solder bumps using a tiered circular illumination technique and SVM. Zhang *et al.* [24] carried on the image analysis based on the non-linear Mumford-Shah model and utilized the SVM for flip chip defect recognition.

In this paper, ultrasonic inspection of flip chips using a 230 MHz transducer was carried out. The time-domain signals and the images of flip chip solder bumps were captured by SAM, normalized cross-correlation (NCC) was used to locate the center of solder bumps for segmenting the flip chip images, and SVM was introduced for flip chip defects inspection. The results demonstrate the feasibility of this approach.

## 2. Theoretical Background

### 2.1. Ultrasonic Inspection

**Figure 1.**
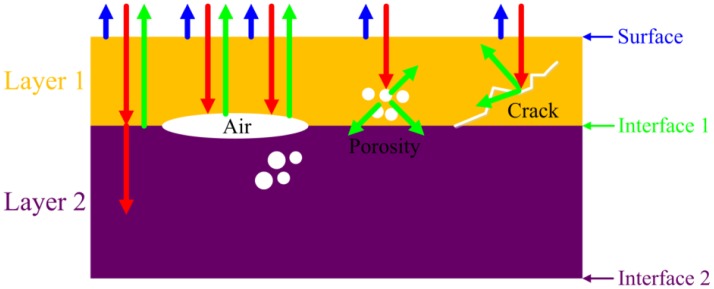
Schematic propagation of the ultrasound through materials with different defects.

Ultrasound is an elastic disturbance that propagates through materials (mainly solids and liquids) at frequencies above 20 kHz [25]. Figure 1 shows the schematic propagation of the ultrasound through materials with different defects. When an ultrasonic wave impinges upon a boundary between different materials with the acoustic impedances denoted by z_2_ and z_1_, some of the energy is reflected and the rest is transmitted. The reflection coefficient *R* and the transmission coefficient *T* are calculated by:

(1) $$R=\frac{\text{Reflected}}{\text{Incident}}=\frac{z_{2}-z_{1}}{z_{2}+z_{1}}$$

(2) $$T=\frac{\text{Transmitted}}{\text{Incident}}=\frac{2z_{2}}{z_{2}+z_{1}}$$

In this work the reflection mode was adopted to detect the defects of the flip chip solder bumps. According to Equation (1), the higher the acoustic impedance mismatch, the stronger the signal reflects.

### 2.2. Principle of NCC

NCC is a fast and efficient method for many machine vision applications. It is used to compute the normalized cross-correlation of the template and the scene by the formula [26]:

(3) $$\gamma \left(u,v\right)=\frac{\sum_{x,y}[f\left(x,y\right)-{\bar{f}}_{u,v}][t\left(x-u,y-v\right)-\bar{t}]}{{\left\{\sum_{x,y}{\left[f\left(x,y\right)-{\bar{f}}_{u,v}\right]}^{2}\sum_{x,y}{\left[t\left(x-u,y-v\right)-\bar{t}\right]}^{2}\right\}}^{0.5}}$$

where *f* is the image, and the sum is over *x*, *y* under the window containing the feature *t* positioned at (*u*,*v*),*t̄* is the mean of the feature and *f̄_u,v_* is the mean of *f*(*x,y*) in the region under the feature.

The advantage of the NCC is that it is less sensitive to linear changes in the amplitude of illumination in the two compared images. Furthermore, the cross-correlation coefficient is confined in the range between −1 and 1, leading to easier setting of the threshold than the cross-correlation.

### 2.3. Principle of SVM

**Figure 2.**
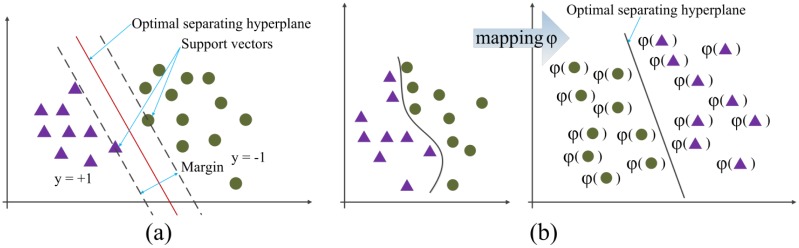
Geometric illustration of SVM. (**a**) Linear SVM; (**b**) Non-linear SVM.

SVM is an important learning method of statistical learning theory, powerful for pattern recognition based on the structural risk minimum principle, in which an optimal separating hyperplane (OSH) is defined to separate two classes. The vectors from the same class fall on the same side of the OSH. The distance from the closest vectors to the OSH is the maximum among all the separating hyperplanes [27]. These vectors are called support vectors. Figure 2a shows a linear SVM. The left side of the OSH is the class labeled by *y* = 1 and the other class on the right side is labeled by *y* = −1. Generally, non-linear problems exist in engineering practices, in which linear SVM is incapable of dealing with them. Then non-linear SVM is introduced to change the linearly inseparable problems into separable ones through mapping the input vectors into a high-dimensional feature space, and new OSH is constructed in the feature space as shown in Figure 2b.

## 3. Flip Chip Defects Inspection

### 3.1. Experimental Procedure

**Figure 3.**
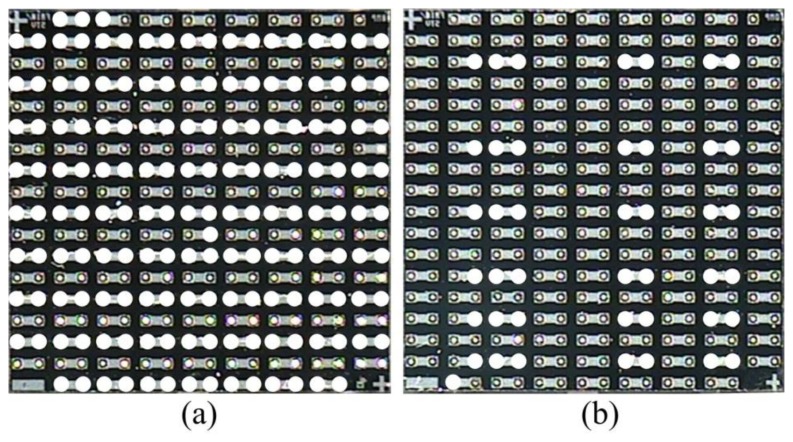
The optical images of the flip chip specimens.

The two flip chip samples obtained from Practical Component are daisy-chain flip chips (FA10-200 × 200, Dummy Components) and non-underfilled for testing. There are 317 solder bumps arranged in 18 rows and 18 columns at 254 μm pitch in each chip. To introduce the defects of missing solder bumps, some solder bumps are removed manually from the flip chips. Figure 3 shows the optical images of the flip chips before bonding captured using an imaging instrument (MC001-YR2010), where the white circles show the distribution of the missing solder bumps in the flip chips.

**Figure 4.**
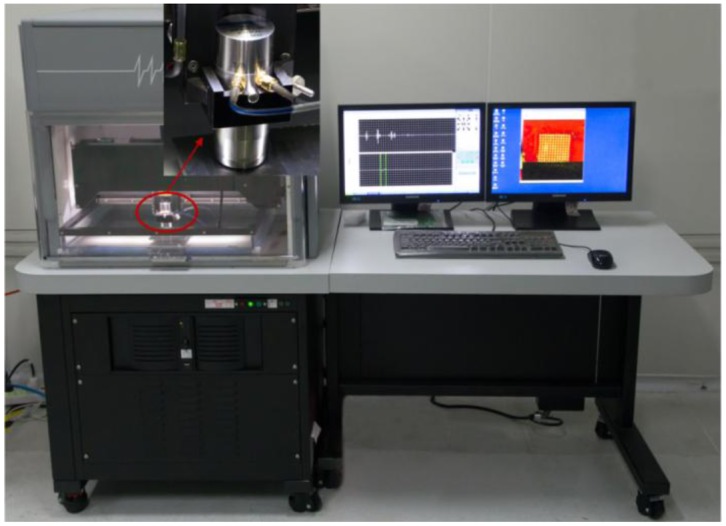
The Scanning Acoustic Microscopy equipment (Sonoscan D9500).

The flip chips were bonded by use of a flexible sub-micron die bonder (FINEPLACER^®^ lambda). After that, an image acquisition system, SAM (Sonoscan D9500) as shown in Figure 4, was used to inspect the flip chips. The flip chip was fixed on the wafer stage and immersed in the de-ionized water which acted as the coupling medium for the acoustic wave propagation. The transducer transmitted signals and scanned the entire flip chip. Then the computer processed the received signals and generated the image of the flip chip. Fifty MHz, 100 MHz and 230 MHz transducers were used to scan the flip chips in our laboratory, and we obtained the optimal ultrasound signals and flip chip images under 230 MHz, because the resolution rises with the increase of the ultrasound frequency. The spot size of the 230 MHz transducer is 0.0318 mm.

**Figure 5.**
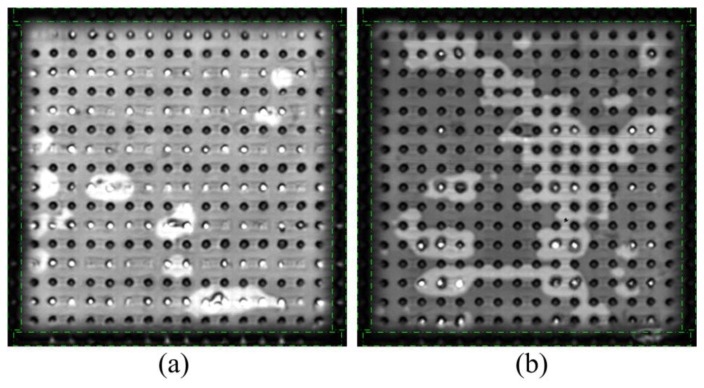

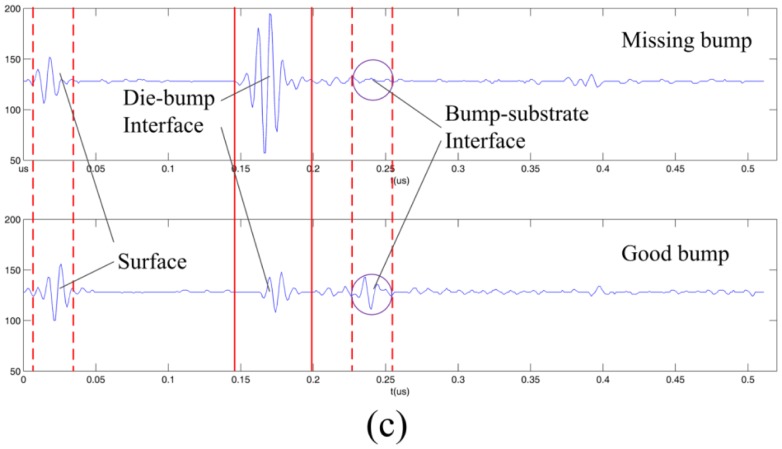
(**a** and **b**) SAM images of the flip chips I and II; (**c**) The typical time-domain signals of good and missing solder bumps.

The SAM images of the flip chips are shown in Figure 5a,b, where the darkness on the brim of the flip chip is caused by the edge effect, making it more difficult to diagnose the solder bumps located on the edges. Here edge bumps were marked by green dash-dotted squares. In order to display the ultrasound features, the typical time-domain signals of the good and missing solder bumps are extracted as shown in Figure 5c. It can be found that as the mismatch of the acoustic impedances under the good bumps is low, and both the die-bump interface and the bump-substrate interface have relatively obvious reflected signals. However, the mismatch under the missing bump is high, so that the die-bump interface has a strong reflected signal as the bump-substrate interface almost has no reflected signal.

### 3.2. Feature Extraction and Pattern Recognition for Flip Chip Diagnosis

The flip chip contains a large number of solder bumps, which correspond to the regions of interest (ROIs) in the SAM images. The ROIs were segmented and one of the good solder bump images was selected manually as the referenced bump image. NCC was employed to obtain the correlation coefficients matrix of the flip chip SAM images and the referenced solder bump image. The peak values in the matrix are located at the center of the solder bump images. Then we obtained all the solder bump images from the flip chip SAM images, got the corresponding time-domain signals, and extracted five features for further classification and recognition.

Let *S* denotes the *p* × *q* matrix associated with the solder bump image and *S*(*i,j*) is the *ij*-th gray entry of *S*. The gray value *G_db_* of the solder-bump image *S_db_* in the die-bump interface is defined by:

(4) $$G_{db}=\sum_{i,j}^{p,q}S_{db}(i,j)$$

The gray value *G_bs_* of the solder-bump image *S_bs_* in the bump-substrate interface is defined by:

(5) $$G_{bs}=\sum_{i,j}^{p,q}S_{bs}(i,j)$$

The interfaces can be recognized based on the analysis of the time-domain signals. In order to calculate the maximum amplitude in each interface, we determine the time range of the die-bump interface from 0.146 s to 0.196 s and the time range of the bump-substrate interface from 0.225 s to 0.252 s, as shown in Figure 5c. The maximum amplitude *A_db_* of the time-domain signals in the die-bump interface is defined by:

(6) $$A_{db}=\underset{0.146<t<0.196}{\max}f(t)$$

The maximum amplitude *A_bs_* of the time-domain signals in the bump-substrate interface is defined by:

(7) $$A_{bs}=\underset{0.225<t<0.252}{\max}f(t)$$

We define contrast *C* by:

(8) $$C\frac{A_{bs}}{A_{db}}$$

Thus, we can represent every solder bump by a vector *F* as defined by:

(9) $$F=\left[G_{db},G_{bs},A_{db},A_{bs},C\right]$$

Next, LIBSVM, developed by Chih-Jen Lin based on the SVM principle [28], is employed for missing bumps classification. Considering the non-linear effects in flip chip defect diagnosis, we choose the RBF kernel function and adopt cross-validation method to determine the parameters *c* and *g*. Other parameters are set to the default values.

## 4. Results and Discussion

**Figure 6.**
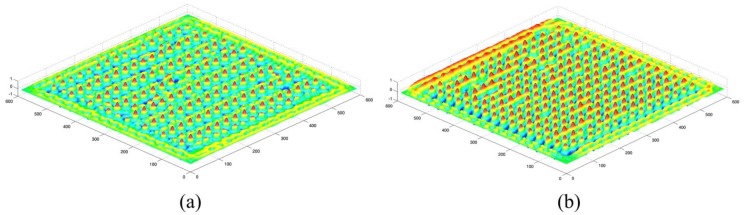
The NCC output matrices of the referenced solder bump image to the SAM images of flip chips I (**a**) and II (**b**).

The NCC output matrices of the SAM images and the referenced solder bump image are figured out as shown in Figure 6, where the peak values correspond to the centers of the solder bumps.

Then we calculated the pitch of two adjacent solder bumps, segmented the solder bumps images, obtained 634 (317 × 2) solder bump images, and acquired the corresponding time-domain signals. Five features were extracted from each solder bump, and we obtained 634 feature data for further analysis. 80 feature data selected randomly from chip-I were normalized for SVM training, and the other dataset (237 feature data from chip-I and 317 feature data from chip-II) were normalized and input to the trained SVM for classification and recognition.

**Figure 7.**
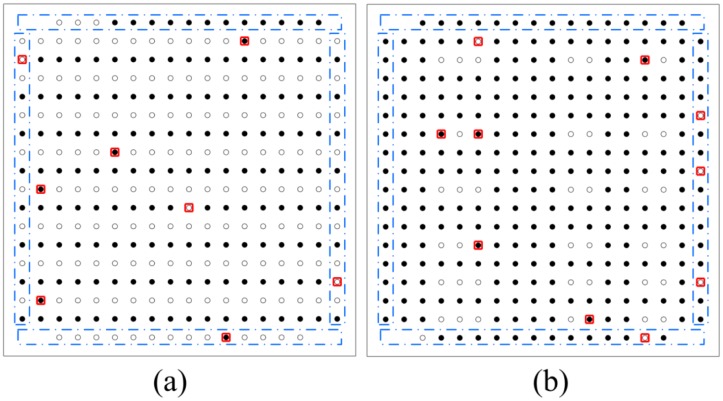
The recognized results of the flip chips.

The classified results are shown in Figure 7 and listed in Table 1, where the solid black spots denote the defect-free solder bumps, the white spots denote the missing solder bumps, and the solder bumps detected incorrectly are marked by red squares. Edge bumps are marked by blue dash-dotted squares. It can be found that there are 18 solder bumps detected incorrectly in total and the averaged error rate is 2.84%. There are eight solder bumps detected incorrectly with the error rate 2.52% in the flip chip I and 10 solder bumps detected incorrectly with the error rate 3.15% in the flip chip II. These results are better than those reported in [21] (the corresponding error rates are 5.99% and 5.68%, and the averaged error rate is 5.84%), all reduced to about the half. Especially, the influences of the edge effect on the recognition become weaker, as only seven solder bumps were detected incorrectly in this work compared with 19 solder bumps detected incorrectly in [21]. Thus, the five characteristics extracted from the solder bump images and the time-domain signals are more reasonable and the classification method using SVM is more effective.

## 5. Conclusions

The robust learning method, SVM, is introduced for ultrasonic inspection of flip chip solder bumps. The diagnosis approach is performed in a sequence of three steps: flip chip SAM imaging and time-domain signal acquisition, feature extraction, and solder bump defect classification and recognition. Experimental investigations have been conducted. The flip chips were bonded by use of a flexible sub-micron die bonder. The SAM images of the flip chips and the time-domain signals were captured using a 230 MHz transducer. Then NCC was adopted to locate the center of every solder bump for segmenting the flip chip images, and five features were extracted. After that, the SVM was used for defects classification and recognition. Two flip chip specimens with 634 solder bumps were detected. There were eight solder bumps detected incorrectly with an error rate of 2.52% in flip chip I and 10 solder bumps detected incorrectly with an error rate 3.15% in flip chip II. Eighteen solder bumps are detected incorrectly in total and the average error rate is 2.84%. The results demonstrate a high recognition rate for missing solder bump inspection. Compared with the work reported in reference [21], the error rates are reduced and the influences of the edge effect on the recognition become weak by using this method. Thus, this diagnosis approach is more effective and can be used for the solder bump inspection in high density packaging. Next, the SAM image capturing needs to be enhanced and more feature extraction methods need to be studied in order to decrease the error rate in classification and recognition.

## Figures and Tables

**Table 1.** The classified results of the solder bumps in the flip chips.

**The Flip Chip**	**The Solder Bumps Detected Incorrectly**	**The Error Rate (%)**
I	8	2.52
II	10	3.15
Total	18	2.84
